# Gene Regulatory Network Interactions in Sea Urchin Endomesoderm Induction

**DOI:** 10.1371/journal.pbio.1000029

**Published:** 2009-02-03

**Authors:** Aditya J Sethi, Robert C Angerer, Lynne M Angerer

**Affiliations:** National Institute of Dental and Craniofacial Research, National Institutes of Health, Bethesda, Maryland, United States of America; University of California, Berkeley, United States of America

## Abstract

A major goal of contemporary studies of embryonic development is to understand large sets of regulatory changes that accompany the phenomenon of embryonic induction. The highly resolved sea urchin pregastrular endomesoderm–gene regulatory network (EM-GRN) provides a unique framework to study the global regulatory interactions underlying endomesoderm induction. Vegetal micromeres of the sea urchin embryo constitute a classic endomesoderm signaling center, whose potential to induce archenteron formation from presumptive ectoderm was demonstrated almost a century ago. In this work, we ectopically activate the primary mesenchyme cell–GRN (PMC-GRN) that operates in micromere progeny by misexpressing the micromere determinant Pmar1 and identify the responding EM-GRN that is induced in animal blastomeres. Using localized loss-of -function analyses in conjunction with expression of *endo16*, the molecular definition of micromere-dependent endomesoderm specification, we show that the TGFβ cytokine, ActivinB, is an essential component of this induction in blastomeres that emit this signal, as well as in cells that respond to it. We report that normal pregastrular endomesoderm specification requires activation of the Pmar1-inducible subset of the EM-GRN by the same cytokine, strongly suggesting that early micromere-mediated endomesoderm specification, which regulates timely gastrulation in the sea urchin embryo, is also ActivinB dependent. This study unexpectedly uncovers the existence of an additional uncharacterized micromere signal to endomesoderm progenitors, significantly revising existing models. In one of the first network-level characterizations of an intercellular inductive phenomenon, we describe an important in vivo model of the requirement of ActivinB signaling in the earliest steps of embryonic endomesoderm progenitor specification.

## Author Summary

In recent years, “gene regulatory networks” (GRNs) have provided integrated views of gene interactions that control biological processes. One of the earliest networks to be activated in the developing zygotes is the one controlling endomesoderm development. In the sea urchin, this network includes several subnetworks that function in adjacent tiers of cells that form the endoderm and mesoderm of the developing embryo. Although classic embryological manipulations have shown that the precursors of the embryonic skeleton induce endomesoderm fate in adjacent cells, the GRNs regulating this interaction are not understood. To investigate these networks, we ectopically activated a GRN that operates in skeletogenic precursors and characterized the responding GRN in neighboring cells, which adopt an endomesoderm fate. By testing the responsiveness of every core factor in the responding GRN, which allowed us to identify a subset that executes the response to the induction, we demonstrated that the signaling molecule, ActivinB, is an essential component of this induction and that its function is physiologically relevant: it is required during normal embryonic development to activate the same GRN that responds to signals from skeletogenic precursors. Furthermore, the network response to ActivinB signaling reveals greater complexity in an additional uncharacterized inductive signal emitted by skeletogenic precursors. Our results thus highlight how interacting GRNs can be used to understand a fundamental signaling process.

## Abstract 3

A classic embryonic induction in sea urchins is described in terms of interacting gene regulatory networks and a signal transduction system that connects them.

## Introduction

Most animal embryos are patterned through a series of cell–cell interactions that shape and refine maternally inherited axial asymmetries. Cells are directed to assume specific fates through the phenomenon of embryonic induction, a process that has been intensely studied by developmental biologists for a number of decades. Understanding the mechanisms of embryonic induction is also of vital clinical importance, especially in the field of regenerative medicine, in which pluripotent stem cells are induced to differentiate into specific lineages of therapeutic interest. The widespread availability of sequenced eukaryotic genomes and microarray-based tools has greatly facilitated large-scale analyses of genes expressed at various times in the course of an embryonic induction. There is now a critical need to understand the regulatory interactions among these gene products that ultimately determine the outcome of the induction process.

By integrating regulatory interactions among large sets of gene products, gene regulatory networks (GRNs) provide uniquely global perspectives on biological and disease processes. Network-based approaches have therefore been adopted to describe *Drosophila* embryonic patterning [1], immune cell function [2], nematode vulval development [3], vertebrate retinal development [4], metabolic pathways [5], and malignant transformations [6]. The sea urchin endomesoderm–gene regulatory network (EM-GRN) was one of the first large-scale integrations of maternal and zygotic regulatory information in early embryonic development [7] and is currently one of the most detailed biological networks in existence [8]. A number of recent studies employing the sea urchin EM-GRN have demonstrated that, in addition to describing the regulatory blueprint underlying embryogenesis, changes in developmental GRN structure can suggest the molecular basis of the evolution of species-specific cell types and body plans across distant phyla [9,10].

An asymmetric localization of nuclear β-catenin is observed in most deuterostome embryos during early stages of development [11–15]. In sea urchin embryos, this anisotropy occurs along the primary (animal–vegetal) axis [13]. The vegetal wave of nuclearization of β-catenin during early cleavage stages is essential for endomesoderm formation, since embryos that lack nuclear β-catenin function develop as primarily ciliated ectoderm without any endomesodermal tissue [13]. These findings provided the initial experimental leverage for constructing the overall sea urchin pregastrular EM-GRN [7]. The constituent subnetworks of the overall EM-GRN include core sets of transcription factors that function cell autonomously and interact within specific blastomeres that eventually differentiate into primary (primary mesenchyme cell–GRN [PMC-GRN]) and secondary mesoderm (secondary mesoderm–GRN) as well as early endoderm (early endomesoderm/endoderm–GRN [E-EM/En-GRN] and late endoderm-GRN) derivatives (Figure 1A and [8]). Whereas regulatory interactions within each component network of the overall EM-GRN have been studied in considerable detail, critical connections between blastomeres, and thus between individual subnetworks, are not completely understood.

**Figure 1:**
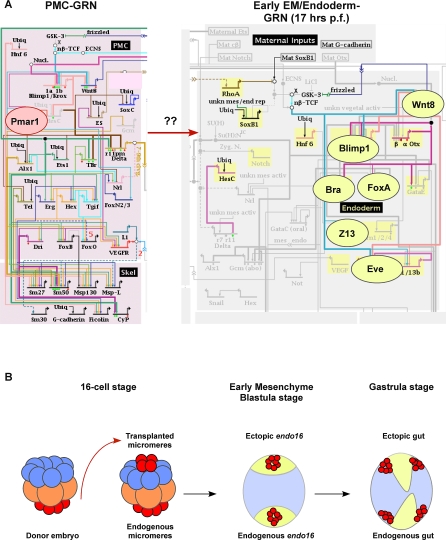
Gene Regulatory Networks Underlying Endomesoderm Induction (A) The micromere determinant Pmar1 (circled in red) activates the PMC-GRN in micromere progeny and is sufficient for micromere-derived endomesoderm-inducing signals. The E-EM/En-GRNs (up to 17 h postfertilization) integrate the regulatory functions of maternal and zygotic core factors that drive the earliest steps of endomesoderm progenitor specification in sea urchin embryos. The zygotically expressed core factors Z13, Eve, Wnt8, Blimp1, FoxA, and Brachyury (Bra) (circled in black) accumulate in presumptive endomesoderm during early developmental stages and could potentially respond to early inductive inputs from micromere descendants. (B) Schematic depicting an experiment that reveals micromere-derived endomesoderm inductive signals, which are sufficient to induce ectopic *endo16* expression and complete archenteron formation in animal blastomeres, and are also necessary for normal vegetal *endo16* expression and timely gastrulation in the sea urchin embryo. The regulatory interactions among these signals and the overall EM-GRN are unknown. GRN diagram is adapted from [8].

One of the earliest inductive phenomena reported in experimental embryology was provided by the vegetal micromeres of the sea urchin embryo. Almost a century ago, Hörstadius reported that micromeres placed at the animal pole of the sea urchin embryo could induce adjacent presumptive ectoderm to differentiate as endodermal vesicles [16]. Several decades later, a molecular analysis confirmed that transplanted micromeres induce expression of the endomesoderm marker *endo16* and eventually formation of a complete secondary archenteron from adjacent presumptive ectoderm (Figure 1B) [17]. Subsequent analyses have provided evidence in the normal embryo for a signal sent by micromeres and their progeny between fourth and sixth cleavages, which is required for normal endomesoderm specification, as monitored by *endo16* expression, as well as for timely gastrulation (Figure 1B) [18]. Micromeres continue to emit archenteron-inducing signals through late cleavage/early blastula stages [19]. Despite several decades of efforts, however, neither the molecular identity(s) of these micromere-originated signals nor their specific regulatory contribution to early endomesoderm specification has been elucidated. Collectively, this constitutes a set of major unanswered questions in echinoderm embryo development.

The β-catenin–dependent determinant Pmar1 is transiently expressed in micromere progeny during the initial stages of specification of this cell lineage [20]. Pmar1 is sufficient for micromere-originated endomesoderm inducing signals and can regulate most aspects of micromere differentiation through activation of the PMC-GRN [21,22]. Pmar1′s ability to activate the PMC-GRN in any blastomere provides a unique opportunity to understand how signaling outputs of the PMC-GRN are interpreted through the regulatory logic of the recipient E-EM/En GRN (Figure 1A).

In this study, we resolve the regulatory connections between the Pmar1-activated PMC-GRN and the E-EM/En-GRN by testing the responsiveness of every zygotically activated core factor of the E-EM/En-GRN to the ectopic expression of the micromere determinant Pmar1. Our studies define a subset of the E-EM/En-GRN that responds to signals from the Pmar1-activated PMC-GRN and show that a significant fraction of the E-EM/En-GRN, including the recently identified Wnt8/Blimp1/β-catenin subcircuit [23], is insensitive to signals emitted by Pmar1-misexpressing blastomeres. We show that the transforming growth factor-β cytokine, ActivinB, is required both in Pmar1-expressing cells that emit *endo16-*inducing signals and in blastomeres that respond to this induction, making it an essential component of both emitting and responding GRNs. Furthermore, we demonstrate that ActivinB function is necessary for activation of the Pmar1-inducible subset of the E-EM/En-GRN in early endogenous endomesoderm development, whereas the Pmar1-unresponsive portion of the E-EM/En-GRN operates in vegetal endomesoderm precursors independently of ActivinB signaling. These key findings strongly suggest that ActivinB has an essential role in endomesoderm specification mediated by micromeres and their progeny and unexpectedly reveal the existence of a new micromere-derived signal(s). Collectively, we provide a critical global view of the regulatory interactions that constitute a classic embryonic induction response.

## Results

### Pmar1-Mediated Respecification of Ectoderm Is Regulated by a Subset of Core Factors within the Overall EM-GRN

Previous studies have shown that the micromere determinant Pmar1, through activation of the PMC-GRN, is sufficient for most aspects of micromere differentiation [20–22]. Pmar1 misexpression is sufficient to confer properties of the micromere/PMC lineage to all blastomeres in the embryo, including the ability to induce *endo16* expression and formation of a complete ectopic gut from adjacent blastomeres [20,21]. Therefore, in order to determine which core factors of the E-EM/En-GRN respond to outputs of the PMC-GRN, we generated clones of cells expressing Pmar1 and GFP under the control of the hatching enzyme (*SpHE*) promoter (Figure 2A), which is active in nonmicromere lineages of the sea urchin embryo during early cleavage and blastula stages [24]. Consistent with previous studies [21], ectopic Pmar1-misexpressing cells ingressed into the blastocoel at the same time as endogenous primary mesenchyme cells. Furthermore, at the mesenchyme blastula stage, *endo16* transcripts accumulated in ectopic nonvegetal blastomeres adjacent to *gfp*-positive, Pmar1-expressing cells, as well as in endogenous endomesoderm progenitors ([21]; see below). Collectively, these observations validate the use of the *SpHE-pmar1* construct to examine ectopic E-EM/En-GRN activation in response to Pmar1-dependent induction signals.

**Figure 2:**
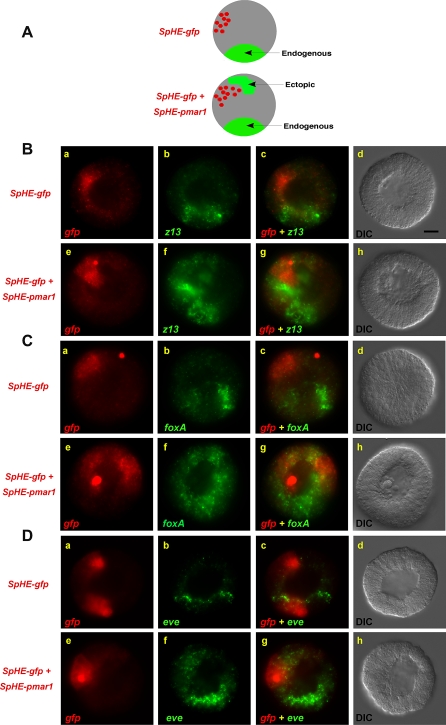
EM-GRN Factors Induced by the Pmar1-Dependent Endomesoderm Induction (A) In all cases shown in (B–D), two-color FISH was used to detect ectopic induction of test transcripts (green) adjacent to *gfp* (red)-mRNA–expressing blastomeres in embryos injected with 0.02 μg/μl *SpHE-gfp* and 0.015 μg/μl *SpHE-pmar1* (e–h in each case) compared to embryos injected with 0.02 μg/μl *SpHE-gfp* alone (a–d in each case). Test transcripts: (B) *z13* expression at 16–18 h p.f. (C) *foxA* expression at 20 h p.f. (D) *eve* expression at 14 h p.f. Embryos shown are representative of over 80% of at least 50 embryos in each of three separate experiments in which *gfp* mRNA was detected in nonvegetal regions. Black scale bar in (B) d represents approximately 20 μm. DIC images in (B) d and h, (C) d and h, and (D) d and h illustrate the absence of detectable developmental defects in injected embryos. *gfp-*expressing cells containing Pmar1 in (C) g and h have adopted a mesenchyme phenotype and ingressed into the blastocoel at 20 h p.f.

The E-EM/En-GRN includes the core regulatory factors, Z13 [25], Blimp1 [26], Wnt8 [27], Eve [28], FoxA [29], and Brachyury [30], all of which are zygotically expressed in endoderm and secondary mesoderm precursors during early cleavage and blastula stages (Figure 1A). We tested for transcripts encoding each of these core factors in cells adjacent to *gfp*-positive, Pmar1-misexpressing cells. At late cleavage and blastula stages, Pmar1 induced ectopic expression of *z13* (Figure 2B, panels e–h vs. a–d)*, foxA* (Figure 2C, panels e–h vs. a–d) and *eve* (Figure 2D, panels e–h vs. a–d) mRNAs within two to three cell diameters of *gfp*-labeled blastomeres. As for the late endomesoderm marker, *endo16*, each of these early genes was ectopically induced in presumptive ectoderm at the same developmental time that its transcripts accumulated in normal vegetal endomesoderm progenitors. In contrast to this set, no ectopic expression of *blimp1* (Figure 3B, panels e–h vs. a–d), *wnt8* (Figure 3C, panels e–h vs. a–d) or *brachyury* (Figure 3D, panels e–h vs. a–d) was detectable in cells adjacent to Pmar1-misexpressing clones at developmental times ranging from 10 to 22 h postfertilization (p.f.), when these transcripts accumulated in presumptive vegetal endomesoderm. These observations strongly suggest that Pmar1-misexpressing blastomeres respecify presumptive ectoderm as endomesoderm by activating only part of the E-EM/En-GRN, consisting of the genes encoding the core regulatory factors Z13, Eve, and FoxA. Ectopic activation of *blimp1*, *wnt8*, and *brachyury* was not detectable, thereby defining a significant part of the E-EM/En-GRN that is not activated by Pmar1-dependent induction.

**Figure 3:**
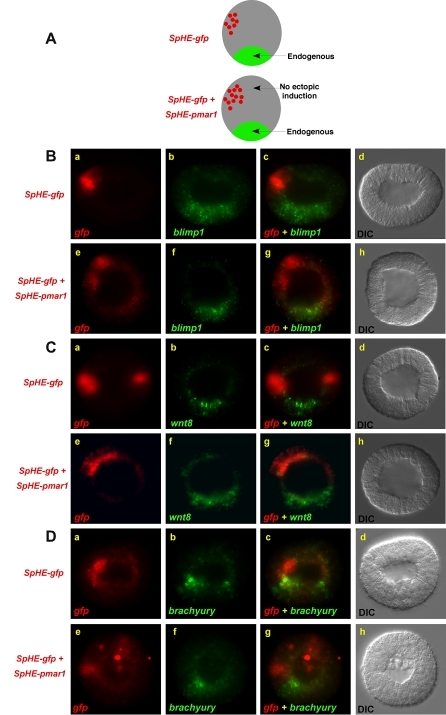
EM-GRN Factors That Are Not Induced by Ectopic Pmar1 Expression (A) In all cases shown in (B–D), two-color FISH showed the absence of ectopic induction of test transcripts (green) adjacent to *gfp*-mRNA–expressing (red) blastomeres in embryos injected with 0.02 μg/μl *SpHE-gfp* and 0.015 μg/μl *SpHE-pmar1* (e–h in each case) compared to embryos injected with 0.02 μg/μl *SpHE-gfp* (a–d in each case). Test transcripts: (B) *blimp1* expression at 14–16 h p.f. (C) *wnt8* expression at 14–16 h p.f. (D) *brachyury* expression at 20 h p.f. Embryos shown are representative of 100% of at least 50 embryos in each of three separate experiments in which *gfp* mRNA was detected in nonvegetal regions. DIC images in (B ) d and h, (C) d and h, and (D) d illustrate the absence of detectable developmental defects in injected embryos. *gfp-*expressing cells containing Pmar1 in (D) panels g and h have adopted a mesenchyme phenotype and ingressed into the blastocoel at 20 h p.f.

### ActivinB Is a Critical Component of Both PMC and EM-GRNs Underlying Pmar1- Dependent Induction

Members of the Nodal/Activin/TGFβ class of transforming growth factor-β cytokines have been implicated in endomesoderm development in vertebrate embryos [31], making them promising candidates for ligands that regulate micromere-dependent endomesoderm induction. The Strongylocentrotus purpuratus genome encodes several candidate TGFβ-related factors that are expressed before the hatching blastula stage [32]. Two of these, Nodal and Univin, are required for oral–aboral specification in the sea urchin embryo, but neither is necessary for endomesoderm formation [33,34]. In addition to these, transcripts encoding an ortholog of ActivinB (GenBank accession number: EU526314) were detected at low levels at developmental times (see below) when micromere progeny emit endomesoderm-inducing signals [18,19]. When ActivinB translation was blocked with either of two different antisense morpholino oligonucleotides (MO), gastrulation was consistently delayed by 12–18 h compared to buffer-injected controls (Figure 4A, panels d and g vs. a), and embryos contained supernumerary pigment cells at 3 d p.f. (Figure 4A, panels e and f, and h and i vs. b and c). Furthermore, we found that expression of *endo16*, the cardinal marker of micromere-mediated ectopic [17] and endogenous [18] endomesoderm induction, was significantly reduced in veg2 progeny at the mesenchyme blastula stage in ActivinB morphants (Figure 4C, panel c vs. a). We observed a similar gastrulation defect (Figure 4B, panels c and d vs. a and b) and inhibition of *endo16* mRNA expression (Figure 4C, panel d vs. b) when we used the small molecule inhibitor, SB-431542 (SB; 5 μM) [35], to block signaling through Activin-like kinase-4/5/7 (ALK-4/5/7) during the first day of embryogenesis. Since ALK4/5/7 is a type I TGFβ receptor that transduces Activin/Nodal/TGFβ-like signals in the sea urchin embryo [34,36], these findings suggest that ActivinB signaling through the SB-sensitive ALK4/5/7 complex is necessary for normal archenteron formation in the sea urchin embryo and could constitute at least part of the signaling machinery required for endomesoderm induction in response to micromere signals.

**Figure 4:**
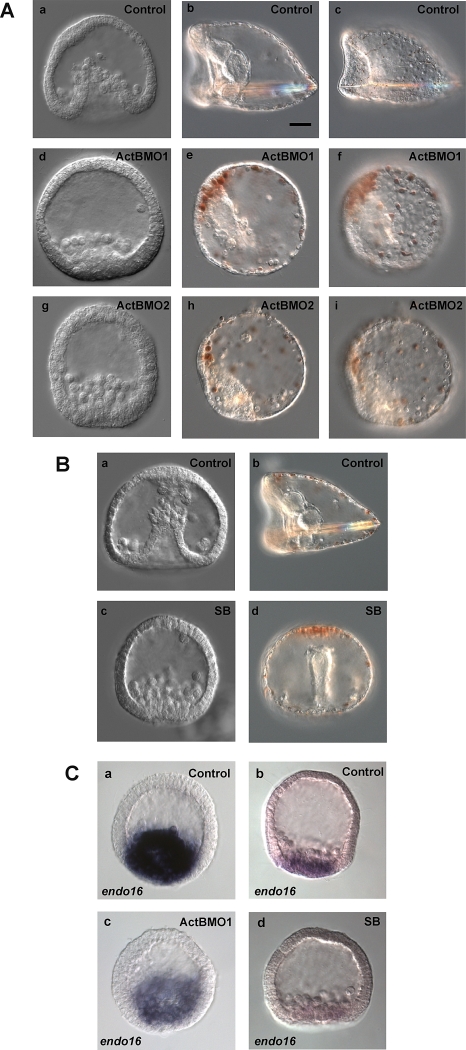
ActivinB-ALK4/5/7 Function Is Required for Timely Gastrulation and Pregastrular *endo16* mRNA Expression (A) Embryos were injected with buffer (Control; a–c) or 1.2 mM ActivinB MO1 (ActBMO1; d–f) or 0.6 mM ActivinB MO2 (ActBMO2; g–i) and photographed at 36 (a, d, and g) and 72 (b, c, e, f, h, and i) h p.f. (B) Embryos were treated with DMSO (a and b) or 5 μM SB-431542 (c and d) for the first day of development and photographed at 36 (a and c) and 72 (b and d) h p.f. Compared to buffer-injected controls ([A] a), gut formation was delayed in embryos injected with ActivinB MO1 ([A] d) and ActivinB MO2 ([A] g). Similarly, gastrulation was delayed in SB-treated embryos ([B] c) compared to DMSO-treated controls ([B] a). (C) Reduced *endo16* mRNA expression was observed at 26 h p.f. in 1.2 mM ActBMO1-injected (c) and 5 μM SB-treated (d) embryos compared to buffer-injected (a) or DMSO-treated (b) controls respectively. Images are representative of the phenotypes observed in over 80% of at least 100 embryos in three separate experiments. Black scale bar in (A) b represents approximately 40 μm.

To determine whether ActivinB is necessary for ectopic endomesoderm induction, we assessed its requirement for *endo16* induction adjacent to blastomeres expressing the micromere determinant Pmar1. Consistent with previous studies [21], in embryos injected with *SpHE-gfp* and *SpHE-pmar1*, *endo16* mRNA accumulated at the mesenchyme blastula stage in ectopic nonvegetal patches of cells adjacent to Pmar1-expressing blastomeres, as well as in its normal vegetal territory (Figure 5A, panels e–h vs. a–d). When ActivinB MO was coinjected with the *SpHE-gfp* and *SpHE-pmar1* constructs, both ectopic and endogenous *endo16* expression were significantly reduced (Figure 5A, panels i–l vs. e–h). This finding identifies ActivinB as an essential component of Pmar1-dependent endomesoderm induction.

**Figure 5:**
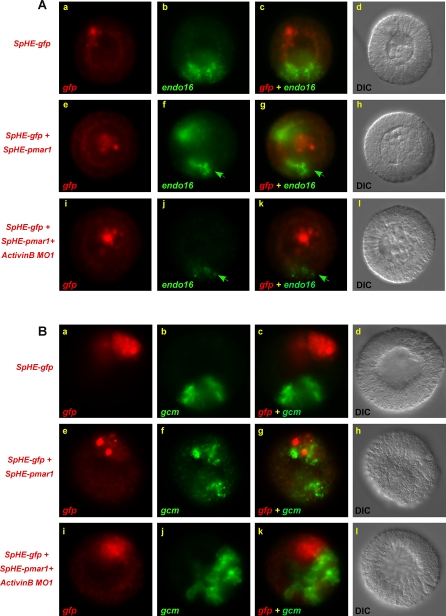
ActivinB Is Required for Pmar1-Mediated Endomesoderm Induction Embryos were injected with either 0.02 μg/μl *SpHE-gfp* or 0.02 μg/μl *SpHE-gfp* and 0.015 μg/μl *SpHE-pmar1*, as indicated to the left of the images. Embryos were analyzed by two-color FISH for *gfp* (red) and either (A) *endo16* (green) at 26 h p.f. or (B) *gcm* (green) transcripts at 18–20 h p.f. The presence of 1.4 mM ActivinBMO1 strongly reduces both ectopic and endogenous *endo16* mRNA levels ([A], i–l vs. e–h, compare arrowheads in [A] j and k vs. f and g for residual endogenous *endo16* expression in the presence of ActivinMO1), but not those of the pigment cell marker, *gcm* ([B] i–l vs. e–h). Embryos shown are representative of more than 80% of a minimum of 50 embryos in each of three separate experiments in which *gfp* was detected in nonvegetal regions of the embryo. DIC images in (A) d and (B) d, h, and l illustrate the absence of detectable developmental defects in injected embryos. *gfp-*expressing cells containing Pmar1 in (A) h and l have adopted a mesenchyme phenotype and ingressed into the blastocoel at 26 h p.f.

Pmar1 is sufficient to activate all known inductive properties of micromeres, and cells misexpressing this determinant express Delta [20], which signals from micromeres through the Notch receptor to induce expression of the pigment cell marker, *gcm*, in neighboring blastomeres (Figure 5B, panels e–h vs. a–d) [37,38]. In contrast to *endo16* induction, both endogenous and ectopic *gcm* induction were unaffected by coinjection of ActivinB MO along with the *SpHE-gfp* and *SpHE-pmar1* constructs (Figure 5B, panels i–l vs. e–h). Thus, ActivinB is specifically required for Pmar1-mediated respecification of ectoderm as endomesoderm and not for micromere Delta-dependent secondary mesenchyme induction [37].

To test whether ActivinB function is required within Pmar1-expressing cells or within the cells that receive signals from them, we microinjected *pmar1* and *gfp* mRNAs into one blastomere of a two-cell embryo and ActivinB MO into either the same blastomere or its sister. When ActivinB translation was inhibited exclusively within the Pmar1-misexpressing cells, induction of ectopic *endo16* mRNA (at 26 h p.f.) in the other half of the embryo was significantly compromised (Figure 6, panels g–j vs. b–e). A small region of residual endogenous *endo16* transcript accumulation persisted in the uninjected embryo half, demonstrating that the morpholino did not diffuse to the progeny of the uninjected blastomere (arrowheads in Figure 6, panels i and j). The descendants of the injected blastomere, on the other hand, adopted a primary mesenchyme cell fate and therefore did not express *endo16*. When ActivinB translation was blocked only in the recipient blastomere, both ectopic and endogenous *endo16* mRNA expression were significantly down regulated in the progeny of that cell (Figure 6, panels k–o vs. a–e). These findings demonstrate that ActivinB is an essential component of the PMC-GRN that is activated in Pmar1-expressing blastomeres, as well as of the responding EM-GRN that operates in neighboring cells. This dual requirement suggests that ActivinB from Pmar1-expressing cells probably activates an autoregulatory loop in recipient cells that is necessary for amplifying its own response, as has been shown for other members of the Nodal/Activin subfamily of TGFβ cytokines [31]. Consistent with its function in both regulatory subnetworks, as well as the ability of all early blastomeres to respond to induction by micromeres, *activinB* transcripts accumulate throughout the embryo at early cleavage and blastula stages of embryogenesis (Figure 7, panels a–c) when micromere-derived endomesoderm inducing signals are sent [16,17,39]. Although Pmar1 induction of early endomesoderm specification requires ActivinB function, ectopic Pmar1 expression does not induce *activinB* mRNA expression (unpublished data). Additional studies will be required to determine the molecular basis of the interaction between Pmar1 and ActivinB.

**Figure 6:**
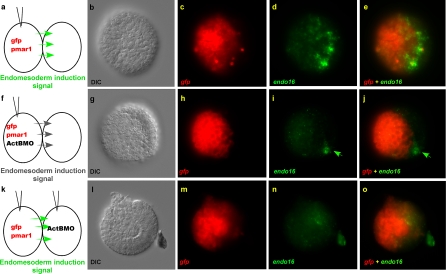
ActivinB Is Required in Cells That Emit and Receive Pmar1-Dependent Induction Signals Individual blastomeres of embryos were injected as diagrammed to the left and analyzed by two-color FISH for *gfp* (red) and *endo16* (green) transcripts. The concentrations of *gfp* mRNA, *pmar1* mRNA, and ActivinB-MO1 used were 0.5 μg/μl, 0.2 μg/μl, and 1.4 mM, respectively. The presence of ActivinB MO1 in Pmar1-expressing cells (f–j) strongly inhibits ectopic and not endogenous (arrowheads in i and j) *endo16* mRNA expression in the uninjected half of the embryo. When ActivinB MO1 is injected exclusively into blastomeres that respond to Pmar1-dependent induction (k–o), both ectopic and endogenous *endo16* mRNA expression are strongly reduced. At the mesenchyme blastula stage shown, Pmar1 (*gfp*)-expressing cells in c, h, m and b, g, l acquire mesenchymal character and ingress into the blastocoel. Images represent the phenotypes observed in 16/19 (a–e), 43/47 (f–j), and 6/6 (k–o) injected embryos.

**Figure 7:**
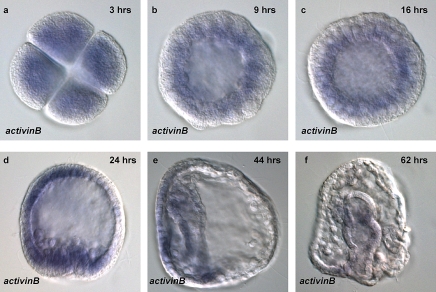
*activinB* mRNA Distribution during Early Sea Urchin Development at the Stages Indicated During early blastula and cleavage stages of development (a–c), low levels of *activinB* transcripts accumulate throughout the embryo. At the mesenchyme blastula stage (d), *activinB* mRNA is expressed in most of the embryo except one side of the ectoderm. At the late gastrula stage (e), *activinB* mRNA expression is detected in the gut and oral ectoderm and in the gut in prism (f) stage embryos.

### ActivinB Signaling in Normal Endomesoderm Development Activates the Pmar1-Inducible E-EM/En-GRN Response

The data described thus far resolve a section of the E-EM/En-GRNs that is activated in response to Pmar1-derived endomesoderm-inducing signals. Furthermore, we demonstrate that Pmar1-dependent ectopic induction of the cardinal endomesoderm marker, *endo16*, requires ActivinB. Previous studies have shown that micromere-derived signal(s) regulate *endo16* accumulation, not only in presumptive ectoderm, but also in the endogenous vegetal plate [17,18]. Therefore, our finding that ActivinB is required for *endo16* accumulation in both ectopically induced and endogenous locations strongly suggests that it mediates micromere-dependent endomesoderm specification in the vegetal plate. Furthermore, as in micromereless embryos, gastrulation is significantly delayed in ActivinB morphants and in embryos treated with SB, which inhibits ALK4/5/7 function [35,36]. ALK4/5/7 function in promoting timely gastrulation is required from early cleavage to blastula stages when micromere progeny signal to overlying veg2 macromere descendants [19] because pulses of 5 μM SB for at least a 4-h interval any time prior to hatching at 18 h p.f. delay gastrulation by 12–18 h, whereas treatment after 18 h p.f. had no effect on the timing of gut development (unpublished data). These results are consistent with a role for ActivinB signaling in early endoderm development.

To determine whether the Pmar1-responsive EM-GRN resolved by the above experiments accurately describes the GRN response to ActivinB signaling in vegetal blastomeres during normal sea urchin development, we characterized the requirement for ALK4/5/7-ActivinB function in pregastrular endomesoderm specification. Whole-mount in situ hybridizations showed that SB treatment significantly inhibits the accumulation of transcripts encoding the core regulatory factors, Z13 (Figure 8A, panel g vs. a), Eve (Figure 8A, panel h vs. b), and FoxA (Figure 8A, panel i vs. c) in the veg2 tier of blastomeres (see fate map in Figure 8C) prior to the hatching blastula stage. The reduction in *z13* and *eve* expression in drug-treated embryos was observed as early as 8 h (∼seventh cleavage) following fertilization. Similarly, a MO-mediated block of ActivinB translation strongly inhibited the expression of *z13* (Figure 8A, panel j vs. d) and *foxA* (Figure 8A, panel l vs. f) mRNAs when assayed at the corresponding stages. We did not detect any difference in the expression of *eve* mRNA in veg2 blastomeres in ActivinB morphants at cleavage stages (Figure 8A, panel k vs. e), suggesting that this transcription factor may be regulated by a TGFβ cytokine other than ActivinB. In contrast, the accumulation of transcripts encoding the remaining members of the E-EM/En-subnetworks, *brachyury*, *blimp1*, and *wnt8*, did not depend on ALK4/5/7 (Figure 8B, panels g, h, and i vs. a, b, and c, respectively) or ActivinB (Figure 8B, panels j, k, and l vs. d, e, and f, respectively) function.

**Figure 8:**
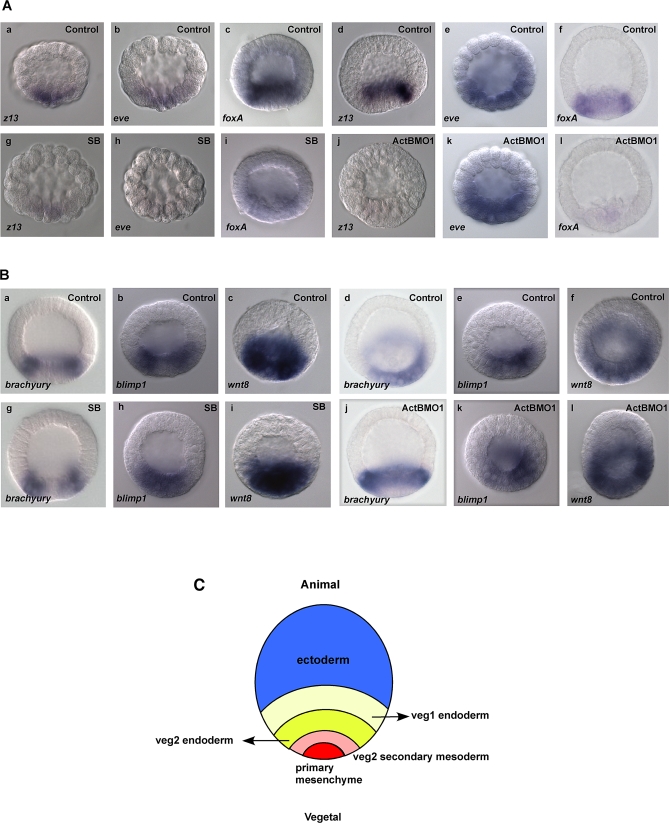
ActivinB-ALK4/5/7 Signaling Activates the Pmar1-Responsive E-EM/En-GRN during Normal Endomesoderm Specification (A) Embryos were treated with either (a–c) DMSO (Control) or (g–i) SB-431542 (SB; 5 μM) and analyzed by WMISH to detect (a and g) *z13* at 8 h , (b and h) *eve* at 8 h, (c and i) *foxA* at 18 h. SB treatment strongly inhibits the accumulation of each of these transcripts in veg2 blastomeres at the stages assayed (g–i) compared to DMSO-treated controls (a–c). The same mRNAs were detected in embryos injected either with buffer (d–f) or ActivinB MO1 (ActBMO1; 1. 2 mM) (j–l) at 14 h (d and j), 10 h (e and k), or 20 h (f and l), respectively. *z13* and *foxA* mRNA expression in veg2 blastomeres was strongly reduced in ActivinB MO1-injected embryos (j and l) compared to buffer-injected controls (d and f) assayed at corresponding stages. At the late cleavage stage, *eve* mRNA expression in the veg2 tier was unaffected by the presence of ActivinB MO1 (k vs. e). (B) Embryos were treated with either (a–c) DMSO or (g–i) SB (5 μM) and analyzed by WMISH to detect (a and g) *brachyury* at 18 h, (b and h) *blimp1* at 14 h, and (c and i) *wnt8* at 14 h. The same mRNAs were detected in embryos injected either with buffer (d–f) or ActBMO1 (1.2 mM) (j–l) at 20 h (d and j), 14 h (e and k), and 14 h (f and l), respectively. The accumulation of each of these transcripts in veg2 blastomeres was independent of ALK4/5/7 (g–i vs. a–c) and ActivinB (j–l vs. d–f) function at the stages assayed. Images in (A) and (B) represent the molecular phenotypes seen in over 80% of at least 50 embryos in three separate experiments. (C) Fate map of blastula stage embryo.

Pigment cells (a secondary mesoderm derivative) were formed at similar times in controls and in embryos lacking ALK4/5/7 or ActivinB function. Consistent with this observation, we found that the first phase of Delta-Notch–mediated secondary mesoderm specification, as assayed by expression of *gcm* mRNA in veg2 progeny [37,38] was independent of ActivinB-ALK4/5/7 activity (Figure 9A, panel e vs. a and panel g vs. c). Similarly, *gataE* was expressed normally in veg2 secondary mesoderm precursors of SB-treated embryos (Figure 9A, panel f vs. b) as well as ActivinB morphants (Figure 9A, panel h vs. d). However, at 34 h p.f., ActivinB morphants contained 40% more *gcm*-positive pigment cell precursors than did buffer-injected controls (Figure 9B, panels d–f vs. a–c), probably due to suppression of early FoxA expression, which represses *gcm* transcription in veg2 endoderm precursors [29]. Thus, similar to its regulatory properties in ectopic Pmar1-mediated induction, normal vegetal activation of the secondary mesoderm (pigment cell)-GRN does not require ActivinB or ALK4/5/7 function.

**Figure 9:**
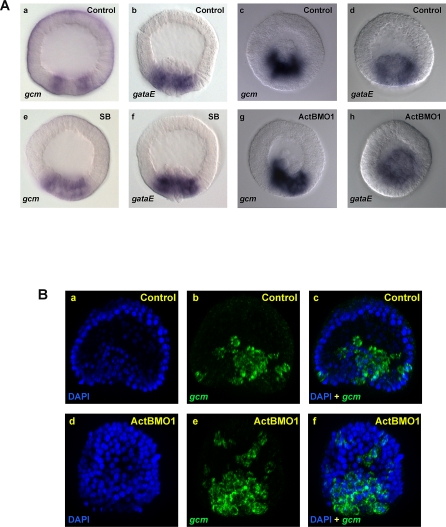
Secondary Mesoderm Specification Is Independent of ActivinB-ALK4/5/7 Function (A) Embryos were treated with either (a and b) DMSO (Control) or (e and f) SB-431542 (5 μM) and analyzed by WMISH to detect (a and e) *gcm* at 18 h and (b and f) *gataE* at 18 h. The same mRNAs were detected in embryos injected either with (c and d) buffer or (g and h) ActivinB MO1 (1.2 mM) at 18 h (c, g, d, and h). The accumulation of each of these transcripts in veg2 secondary mesoderm precursors was independent of ALK4/5/7 (e and f vs. a and b) and ActivinB (g and h vs. c and d) function at the stages assayed. All images are representative of the molecular phenotypes observed in more than 80% of a minimum of 50 embryos in each of three separate experiments. (B) At 34 h p.f., (a–c) buffer-injected controls contain 29 *gcm-*mRNA expressing (*n* = 5 embryos examined) cells (green in b and c) compared to 40 (*n* = 5 embryos examined) such cells (green in e and f) in embryos injected with (d–f) 1.2 mM ActivinB MO1. Nuclei are stained with DAPI (blue).

By the late mesenchyme blastula stage, *z13* and *foxA* transcripts accumulate in veg1 derivatives in addition to veg2 descendants (see fate map in Figure 10B) and the corresponding proteins are essential elements of the late endoderm-GRN. Unlike their earlier expression in the veg2 tier, this later phase of expression does not depend detectably on ALK4/5/7 or ActivinB function (Figure 10A, panel e vs. a, f vs. b, g vs. c, and h vs. d). This finding is consistent with the fact that SB treatment after hatching does not hinder gastrulation and demonstrates that ActivinB signaling through the SB-sensitive ALK4/5/7 complex regulates core elements exclusively within the E-EM/En-GRNs, which function in veg2 blastomeres overlying the micromeres.

**Figure 10:**
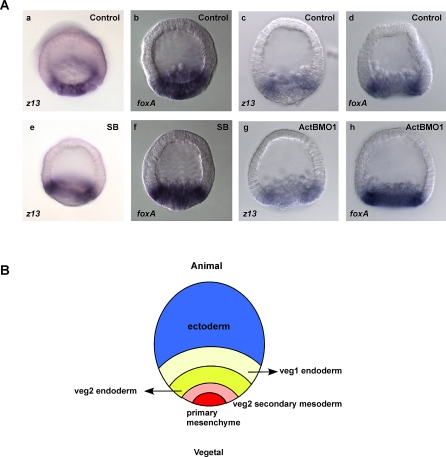
The Late Endoderm-GRN Is Independent of ActivinB-ALK4/5/7 Signaling (A) Embryos were treated with either (a and b) DMSO (Control) or (e and f) SB-431542 (5 μM) and analyzed by WMISH to detect (a and e) *z13* at 26 h and (b and f) *foxA* at 26 h . The same mRNAs were detected in embryos injected either with (c and d) buffer or (g and h) ActBMO1 (1.2 mM) at 26 h (c, g, d, and h). The late accumulation of each of these transcripts in vegetal blastomeres was independent of ALK4/5/7 (e and f vs. a and b) and ActivinB (g and h vs. c and d) function at the stages assayed. Images are representative of the molecular phenotypes observed in more than 80% of a minimum of 50 embryos in each of three separate experiments. (B) Fate map of blastula-mesenchyme blastula stage embryo.

The findings described above demonstrate that the gastrulation defect observed in SB-treated embryos and ActivinB morphants can be entirely attributed to compromised early specification of veg2 endomesoderm progenitors. Interestingly, the GRN response to ActivinB-ALK4/5/7 signaling in normal pregastrular endomesoderm development is almost identical to that of ectopic Pmar1-driven induction. Collectively, these observations strongly suggest that a micromere-derived, ActivinB-dependent signal is necessary for early veg2 endomesoderm progenitor specification through the outputs of a discrete set of core factors of the E-EM/En subnetworks.

### Veg2 Endomesoderm Progenitors Receive Multiple Signals from Micromere Progeny during Early Cleavage

Previous studies have shown that micromeres emit signals during early cleavage stages that clear the β-catenin antagonist SoxB1 from veg2 macromere progeny, a process that has been suggested to be important for the activation of the E-EM/En-subnetworks [21,27,40]. Similarly, when transplanted to animal locations, micromeres also clear SoxB1 protein from surrounding ectoderm cells during their respecification as endomesoderm [21]. Pmar1 misexpression is sufficient to induce SoxB1 down-regulation in adjacent blastomeres in both animal and vegetal contexts [21], and it has been proposed that this clearance occurs in response to the same Pmar1-dependent endomesoderm induction signal that activates *endo16* expression in adjacent blastomeres [21,27]. Since ActivinB is an essential component of this Pmar1-derived induction signal and regulates both ectopic and endogenous *endo16* accumulation, we examined its role in clearing SoxB1 from veg2 secondary mesoderm progenitors. If SoxB1 clearance requires ActivinB, then SoxB1 should persist at high levels in secondary mesenchyme of ActivinB morphants. Surprisingly, however, SoxB1 clears normally from this region, which is marked by *gcm* mRNA accumulation (Figure 11A, panels d–f vs. a–c). We conclude that ActivinB and an unidentified SoxB1-clearing signal are separate, early outputs of micromeres.

**Figure 11:**
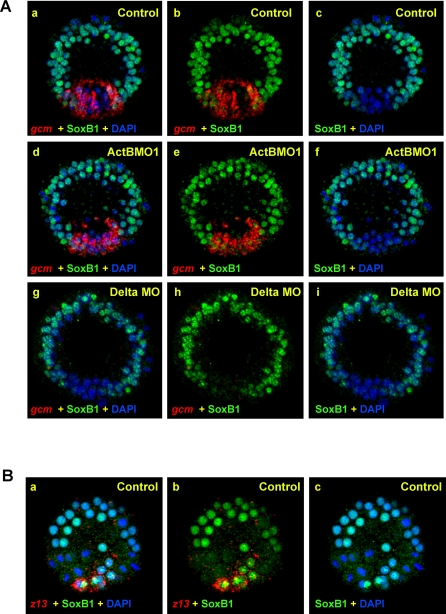
Pmar1-Responsive Endomesoderm Specification Is Independent of the Micromere Signal That Clears Vegetal SoxB1 (A) SoxB1 protein (green), *gcm* mRNA (red), and DAPI nuclear staining (blue) at 18 h p.f.: (a–c) buffer-injected controls; (d–f) 1.2 mM ActivinB MO1–injected embryos; and (g–i) 0.15 mM Delta MO-injected embryos. Images represent the phenotype observed in over 80% of at least 50 embryos in each of three separate experiments. SoxB1 protein clears normally from veg2 secondary mesoderm precursors in ActivinB (d–f vs. a–c) and Delta (g–i vs. a–c) morphant embryos. (B) (a–c) SoxB1 protein (green), *z13* mRNA (red), and DAPI nuclear staining (blue) at 8 h p.f. in normal embryos showing high levels of SoxB1 protein in nuclei of *z13*-expressing cells at the cleavage stage of development. The optical section is slightly oblique with respect to the animal-vegetal axis, and *z13*-expressing cells are at the bottom.

The specific role of SoxB1 clearance in the EM-GRN remains to be elucidated. Although SoxB1 clearance is indicative of micromere signaling, it is gradual and not completed in veg2 progeny until the early mesenchyme blastula stage, after initial veg2 specification has occurred [41,42]. Consistent with this observation, we found that at cleavage stages, genes encoding the Pmar1-responsive endomesoderm core factors, *z13* and *eve*, are expressed in blastomeres that contain high levels of SoxB1 (*z13* shown in Figure 11B; *eve* data not shown), confirming that micromere-mediated early veg2 endomesoderm specification does not require SoxB1 clearance. This is also the case in presumptive endoderm since endoderm-specific genes are expressed normally when the later phase of micromere-dependent SoxB1 clearance is prevented [40]. Furthermore, although endogenous SoxB1 limits nuclear β-catenin activity and SoxB1 misexpression strongly antagonizes it, SoxB1 morphants have greatly *reduced* endoderm and fail to gastrulate [42], demonstrating that some level of SoxB1 is required for normal endoderm development. To test whether down-regulation of SoxB1 might instead be a *consequence* of early specification, we blocked Delta-mediated secondary mesoderm specification by use of a Delta-MO. This approach inhibited *gcm* expression, as expected [37,38], but had no effect on the extent of SoxB1 clearance from veg2 blastomeres (Figure 11A, panels g–i vs. a–c). Thus, micromere-dependent SoxB1 clearance occurs normally when at least two Pmar1-mediated veg2 specification signals (ActivinB and Delta) are blocked. We conclude that the signal for SoxB1 down-regulation, ActivinB, and Delta make largely separate Pmar1-dependent contributions to the development of endomesodermal tissues within the veg2 tier of blastomeres.

## Discussion

This study describes one of the first systematic demonstrations of the developmental GRNs activated in blastomeres that emit and respond to embryonic endomesoderm induction signals. We ectopically activated the Pmar1-dependent PMC-GRN in nonvegetal cells and demonstrate that adjacent blastomeres are specified as endomesoderm through a responding GRN that includes genes encoding the core factors Z13, Eve, and FoxA. In contrast, a significant part of the sea urchin EM-GRN, consisting of the regulatory outputs of Wnt8, Blimp1, and Brachyury, is not activated through Pmar1-mediated signals. Two members of this Pmar1-insensitive set, Wnt8 and Blimp1, have recently been proposed to constitute a self-sustaining *cis*-regulatory subcircuit that maintains nuclearization of β-catenin in endomesoderm progenitors [23]. Since ectopic Pmar1-misexpressing cells do not detectably induce *wnt8* or *blimp1* expression in adjacent animal blastomeres, our work implies that these responding cells are specified as endomesoderm without accumulating β-catenin in their nuclei. This conclusion is consistent with previous studies demonstrating the absence of detectable nuclear β-catenin in animal blastomeres specified as endomesoderm through ectopic micromere induction signals [13].

Using *endo16* expression, the molecular hallmark of micromere-mediated endomesoderm specification [16,17,43], we show through localized loss-of-function analyses that the TGF-β cytokine, ActivinB, is required in both Pmar1-expressing cells that send endomesoderm-inducing signals as well as in the responding EM-GRN in blastomeres that transduce this signal. The requirement for ActivinB in Pmar1-dependent endomesoderm induction strongly suggests that it plays a similar essential role in endomesoderm formation in response to signals from ectopic micromeres, a phenomenon first described almost 70 y ago in the sea urchin embryo [16]. ActivinB signaling through ALK4/5/7 during early cleavage and blastula stages also is necessary for endogenous vegetal activation of almost the entire E-EM/En-GRN induced by ectopic Pmar1 expression. This critical finding, along with the fact that, like micromere progeny, ActivinB is required for normal *endo16* expression in veg2 endomesoderm progenitors, and for timely gastrulation, strongly suggests that this TGFβ ligand plays an essential role in early micromere-dependent endomesoderm specification.

In contrast to the Pmar1-dependent EM-GRN factors, ActivinB is not required for normal vegetal expression of any of the Pmar1-insensitive EM-GRN core factors. Furthermore, both the Delta-dependent secondary mesoderm–GRN and the late endoderm-GRN (17 to 30 h p.f.) that is subsequently activated in veg2 and veg1 macromere progeny are independent of ActivinB function. Thus, the primary cellular targets of ActivinB signaling during pregastrular endomesoderm development are the more vegetal (veg2) macromere derivatives. The exact state of specification of these cells in ActivinB morphants is not clear, but some of them transfate to pigment cells, presumably as a result of reduced FoxA activity [29]. Whether any of these cells adopts other mesodermal fates is not yet known. If indeed they do not contribute to definitive endoderm, then the gut that eventually forms would arise from veg1 progeny that are specified much later than veg2 derivatives [44], through the regulatory outputs of the ActivinB-independent late endoderm-GRN. Alternatively, if veg2 endoderm progenitors in ActivinB morphants do not acquire other secondary mesoderm fates, then they probably participate in gut formation. In this case, either they eventually express all of the core EM-GRN factors through micromere-independent mechanisms or, in a later vegetal developmental context, the Wnt8/Blimp1/nuclear β-catenin subcircuit [23] is sufficient to support this process. Either scenario would require considerable regulatory cross talk between GRN components in order to achieve threshold concentrations of critical core factors that drive definitive endoderm development.

Recent models of pregastrular development in echinoderm embryos have proposed that a single micromere signal specifies veg2 macromere progeny as *endo16*-expressing endomesoderm progenitors and also causes the gradual clearance of the β-catenin antagonist, SoxB1, from nuclei of these blastomeres [21,27,40]. This is an attractive model because it invokes a causal linkage between activation and maintenance of the EM-GRN that depends on nuclear β-catenin accumulation and removal of an antagonist. However, the results of several experiments presented here argue strongly that micromere-mediated early endomesoderm induction and micromere-regulated SoxB1 removal are independent processes. First, SoxB1 down-regulation occurs normally in embryos lacking the early endomesoderm-inducing (*endo16*-inducing) signal ActivinB. Second, SoxB1 clearance is not required for activation of the early EM-GRN because macromere progeny express early Pmar1-responsive core factors in this subnetwork before SoxB1 protein levels are detectably reduced in nuclei of the same cells. Third, ectopic micromere-mediated EM-GRN activation and complete archenteron formation occur in cells that lack detectable nuclear β-catenin [13] but still clear SoxB1 [21]. Our findings are consistent with other studies showing that endoderm specification can occur without SoxB1 clearance [40]. The first phase of SoxB1 clearance in presumptive secondary mesoderm also does not depend on other known micromere signals, because it occurs normally in either Delta (this work) or Wnt8 morphants [27]. Therefore, although SoxB1 clearance depends on an early micromere signal(s), it is a gradual process that is not completed until the mesenchyme blastula stage and relies on an unknown pathway(s) distinct from the one that regulates *endo16* induction through ActivinB (Figure 12). Therefore, we favor the view that fine-scale patterning of endoderm and mesoderm in different blastomere tiers probably requires micromere-dependent regulation of the level and duration of SoxB1 expression, but early micromere-mediated endomesoderm specification is independent of this process (Figure 12).

**Figure 12:**
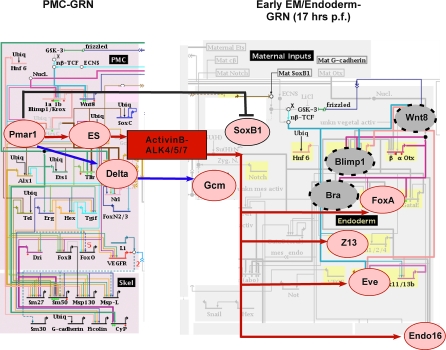
Pregastrular EM-GRN Subnetworks Mediating Micromere-Mediated Endomesoderm Induction The micromere determinant Pmar1 activates the PMC-GRN and transmits endomesoderm-inducing signals to macromere descendants. The Pmar1-responsive genes *z13*, *foxA*, *eve* (and the cardinal endomesoderm marker, *endo16*) define a responding EM-GRN (red arrows leading to factors circled with solid red lines) that is activated in animal blastomeres. The Pmar1-unresponsive genes *wnt8*, *blimp1*, and *brachyury* (Bra) (circled with hatched black lines) are dispensable for ectopic Pmar1-mediated endomesoderm induction, which probably occurs without inducing ectopic nuclearization of β-catenin in animal blastomeres. In both animal and vegetal contexts, ActivinB is necessary for Pmar1-activated endomesoderm induction and eventual *endo16* expression (shown at bottom right of diagram) and is an essential component of the PMC-GRN and E-EM/En-GRNs. ES refers to an early signal, thought to depend on Pmar1, that is sent from micromeres starting after fourth cleavage stage, which, like ActivinB, is required for *endo16* expression and timely gastrulation. ActivinB-independent Pmar1-derived Delta signaling (blue arrow) specifies pigment and blastocoelar cell fate within secondary mesoderm precursors and regulates *gcm* expression in veg2 descendants (the SMC-GRN core factor, Gcm, shown in the diagram is not an element of the E-EM/En-GRNs). A third, unknown Pmar1-mediated signal clears SoxB1 (black line leading to factor circled with solid black line) from endomesoderm precursors and, contrary to previous models of pregastrular endomesoderm development, is independent of micromere-mediated specification of these blastomeres. GRN diagram is adapted from [8].

This work is the first report, to our knowledge, of the requirement of ActivinB in the earliest steps of endomesoderm specification in any developmental model system and of the involvement of a TGFβ cytokine of the Activin/Nodal/TGFβ class in endomesoderm formation in an invertebrate embryo. Activin was initially proposed to be a mesoderm-inducing agent because exogenous Activin can induce mesoderm in amphibian animal cap explants [45]. Activin can also direct endoderm differentiation in human and mouse embryonic stem cells, which, interestingly, transit through an endomesoderm-like primordium prior to expressing markers of definitive endoderm [46,47]. Recent studies in which ActivinB was knocked down with MOs in amphibian embryos suggest that it does play some role in axial mesoderm formation, possibly by regulating convergent extension movements of gastrulation through the activities of other mesoderm-inducing factors [48] and/or the timing of cell cycle transitions in involuting dorsal axial mesoderm [49]. However, even though ActivinB is expressed during early development of amphibian embryos [50], there is, at present, no evidence suggesting that it functions in early endomesoderm specification per se*.* By analyzing the EM-GRN response to ActivinB, we show that it is exclusively required for the earliest steps of endomesoderm specification, eventually leading to the formation of definitive endoderm and timely gastrulation in the sea urchin embryo. This work, therefore, provides a critical in vivo model of the requirement of ActivinB function in early embryogenesis. Furthermore, the remarkable developmental plasticity of the sea urchin embryo allows us to demonstrate that ActivinB is the cardinal primary mesenchyme-derived signal that can activate an ectopic endomesoderm GRN. We thus describe an important experimental paradigm that elucidates the GRNs that drive endogenous and ectopic endomesoderm induction.

By defining major regulatory connections between individual networks of the overall sea urchin EM-GRN, we have built a new framework for future studies of endomesoderm specification. In the sea urchin embryo, endomesoderm development is highly regulative and eventually occurs even in the absence of micromeres [18,43]. Similarly isolated macromeres also express endoderm markers [51]. Our identification of a Pmar1-insensitive set of core regulatory factors may significantly inform future work addressing how the network responds in these situations, potentially leading to an understanding of the molecular basis of such regulative endomesoderm development. Our work also postulates the existence of an uncharacterized micromere signal that is emitted during early cleavage stages to clear the β-catenin antagonist SoxB1 from endomesoderm precursors. Identifying this signal and its GRN properties will be necessary to achieve a comprehensive understanding of pregastrular development in echinoderm embryos. We show that endomesoderm is ectopically induced through the regulatory outputs of a set of Pmar1-responsive EM-GRN core factors, making it important to understand how this core set activates and stabilizes downstream GRN circuits. Since this Pmar1-responsive GRN potentially drives formation of a complete archenteron without detectable nuclear β-catenin, it is also conceivable that additional uncharacterized β-catenin–independent GRNs exist. Interestingly, recent studies have demonstrated the existence of such a β-catenin-independent JNK/Axin dorsalization pathway in zebrafish embryos (e.g., [52]). The sea urchin embryo, with its well-known developmental plasticity, would be an ideal system for characterizing such nascent GRNs and examining their interactions with the currently understood endomesoderm regulatory networks.

## Materials and Methods

### Embryo cultures and inhibitor treatments.

Adult sea urchins (S. purpuratus) were obtained from Marinus Scientific (Garden Grove, California) or The Cultured Abalone (Goleta, California). After removal of fertilization envelopes, embryos were cultured at 15 °C in artificial seawater (ASW) at fewer than 300 embryos/ml of ASW. A stock solution of the ALK4/5/7 inhibitor SB-431542 (Tocris Biosciences) in dimethylsulfoxide was added to cultures to a final concentration of 5 μM. Inhibitor treatments were terminated by pipetting embryos through five ASW-containing 60 × 15-mm Petri dishes. Only cultures in which more than 90% control embryos were morphologically normal were used.

### Microinjections.

Fertilized S. purpuratus eggs in 60 × 15-mm Petri dishes coated with 1% (w/v) protamine sulfate were microinjected using an Eppendorf Femtojet-Injectman NI2 micromanipulator attached to a Leica inverted microscope. Microinjections were performed in PABA-ASW followed by three rinses in ASW. Injection solutions containing 25% glycerol and 10 mM Tris-HCl (pH 8.0) were filtered through 0.22 μM PVDF filters (Millipore) and briefly incubated at 50 °C before loading to minimize clogging within the injection needles (Femtotips I; Eppendorf). For single-blastomere injections, embryos were cultured in ASW until 40 min after the first cell division and rinsed four times with ice-cold calcium-magnesium–free sea water (CMFSW). Embryos were microinjected within 10 min of the last CMFSW rinse, washed four times with ice-cold ASW, and cultured to the desired developmental stage at 15 °C.

### Construct preparation, RNA synthesis, 5′ RACE, and morpholino sequences.

The *SpHE-GFP* construct contains the *SpHE* minimal promoter sequence (from −310 to +96, [24]) inserted between the EcoRI and SalI sites of pGreenLantern (pGL). For the *SpHE-pmar1* construct, the GFP fragment from *SpHE*-*GFP* was replaced with the Pmar1 coding region (GenBank accession number: AF443277) and the first six base pairs upstream of its initiation codon. Both constructs were linearized with PstI prior to microinjection.

*pmar1* and *gfp* mRNAs were synthesized from linearized pCS2^+^-*pmar1* and pCS2^+^-*gfp* templates using the mMessage mMachine kit (Ambion). RNA concentrations were measured by spectrophotometry, and RNA integrity was verified by electrophoresis through agarose-formaldehyde gels prior to microinjection.

To determine the 5′-end of *activinB* mRNA, RACE (rapid amplification of cDNA ends)-ready cDNA was synthesized from 1 μg of DNA-free total RNA extracted from eggs using the SMART RACE cDNA synthesis kit (BD Biosciences) per the manufacturer's instructions. A touchdown-PCR protocol recommended by the manufacturer was used to amplify an *activinB* 5′ RACE product with the *activinB* gene–specific reverse primer, (5′-TCACGAACACCCACAACTCAAGATCCTCATTTC-3′) and the kit's SMART oligonucleotide forward primer mix. RACE products were cloned into PGEM T-Easy (Promega), and 20 clones were sequenced completely in both directions to determine the 5′-end of *activinB.* The *activinB* mRNA sequence has been deposited in GenBank with the accession number of EU526314.

### Morpholino-substituted antisense oligonucleotide (MO) sequences.

Sequences were as follows: SpActivinB MO1: 5′-gcaggtacagagcttctcggtcaac-3′; SpActivinB MO2: 5′-TATCAAGCTGGCCCACGACATGAAA-3′; and Delta MO: 5′-GCCGATCCGTTATTCCTTTCTTATC-3′.

MOs were dissolved in 100 μl of nuclease-free water to give 3 mM stock solutions, which were diluted to the concentrations indicated in the figure legends.

### Whole-mount in situ hybridizations (WMISH) and immunostains.

Embryos were fixed in 4% paraformaldehyde-ASW containing 10 mM MOPS (pH 7.0) and 0.1% Tween-20 for 1 h at room temperature (RT) in 96-well flat-bottomed plates. Hybridization and detection procedures using colorimetric alkaline phosphatase-conjugated digoxigenin antibodies were carried out as described previously [53].

For single-color fluorescent in situ hybridizations (FISH) using a digoxigenin-conjugated RNA probe and tyramide signal amplification (TSA-Plus kit; PerkinElmer), the above procedure was modified as follows. After posthybridization washes, embryos were incubated for 1 h at RT in a blocking buffer consisting of 10% normal goat serum and 5 mg BSA/ml in MOPS wash buffer [53], followed by 30 min at RT in horseradish peroxidase (POD)-conjugated anti-digoxigenin antibody (Roche) (1:1,500) in blocking buffer. Embryos were washed eight times over the course of 2 h at RT with MOPS wash buffer. Hybrids were detected with a 1:100 dilution of TSA-stock solution (FITC TSA-Plus kit or Cy3-TSA Plus kit; PerkinElmer) in diluting buffer at RT for 6–8 min, followed by five washes in MOPS wash buffer at RT.

For dual-color FISH with fluorescein- and digoxigenin-conjugated RNA probes, after the blocking step, embryos were incubated in POD-conjugated anti-fluorescein antibody (Roche) (1:750) at RT overnight, followed by eight washes with MOPS wash buffer. Duplexes were detected by incubating embryos in Cy3 TSA (1:100 dilution) for 6–8 min, followed by five washes with MOPS wash buffer. The POD activity of the bound anti-fluorescein antibody was quenched with 0.1% (w/v) sodium azide in MOPS wash buffer for 30 min at RT, followed by six washes with MOPS wash buffer for a total of 60 min. Digoxigenin-labeled transcripts were detected subsequently using FITC-TSA as described above.

The dual-labeled FISH procedure was modified to allow hybridization of probes to endogenous transcripts and mRNA synthesized from injected *SpHE* promoter-containing plasmids but to exclude formation of hybrids with plasmid DNA [54]. To prevent DNA denaturation during hybridization, the formamide concentration was reduced to 50% and the hybridization temperature lowered to 45 °C. After 4–7 d of hybridization, unhybridized probe was removed with five washes in MOPS wash buffer at 50 °C for 3 h [54]. Residual binding of the probe to DNA was eliminated by two washes in standard hybridization buffer (70% formamide) for 30 min at 50 °C.

For single-color FISH and SoxB1 immunostaining double labeling, FISH was performed first as described above. Embryos were incubated overnight at 4 °C with SoxB1 primary antibody that had been filtered by centrifugation through a 0.22-μm PVDF mesh (Millipore) and diluted 1,000-fold in a buffer containing 5% normal lamb serum, 1× phosphate-buffered saline (PBS), and 0.05% Tween-20 (PBST blocking buffer). After five washes for a total of 1 h at RT with PBST wash buffer (1× PBS, 0.05% Tween-20), bound primary antibody was detected with goat anti-rabbit Alexa-488 secondary antibody (1:750; Invitrogen) in PBST blocking buffer for 1 h at RT.

### Image processing.

Images were captured on a Zeiss Axiovert200 inverted microscope equipped with an Axiotome axial tomography device and differential interference contrast (DIC) optics in Axiovision Release 4.6. Images were processed in Adobe Photoshop 8.0 and Canvas X. Z-stack composites were generated using Imaris Release 5.7.2 (Bitplane).

## Abbreviations

ALK: Activin-like kinase
E-EM/En-GRN: early endomesoderm/endoderm–gene regulatory network
EM-GRN: endomesoderm–gene regulatory network
FISH: fluorescent in situ hybridization
GRN: gene regulatory network
MO: morpholino oligonucleotide
p.f.: postfertilization
PMC-GRN: primary mesenchyme cell–gene regulatory network
SB: SB-431542
WMISH: whole-mount in situ hybridization
